# A minimally invasive, low-stress method for serial blood collection in aging mice

**DOI:** 10.1080/20010001.2019.1647400

**Published:** 2019-07-26

**Authors:** Marianne Bjorner, Lida Zhu

**Affiliations:** Department of Comparative Medicine, School of Medicine, University of Washington, Seattle, WA, USA

**Keywords:** Blood collection protocol, aging mice, hematology, white blood cell differentiation

## Abstract

Hematologic analysis is an efficient and valuable tool for real-time health monitoring and immune analysis in mouse aging studies. However, many frequently used blood sampling techniques in mice are incompatible with continuous monitoring with increasing age, as they may involve anesthesia, cause severe stress, or require a high volume of blood. This technical report describes a convenient relatively noninvasive procedure for counting white blood cells in C57Bl/6 mice by an optimized tail blood collection method followed by Wright-Giemsa and Türk staining. This technique can be performed on unanesthetized mice in less than 1 min with minimal stress using only a few microliters of blood. White blood cell analysis can include but is not limited to total and differential white blood cell count and cell morphology. The low blood volume collected is ideal for aging mice in longer-term experiments requiring regular and continuous monitoring.

## Introduction

Alterations in peripheral white blood cell counts can be triggered by a plethora of diseases, including diabetes, cancer, and sleep deprivation[1]. Monitoring these changes in mouse models has important implications for studying age-related disease [2] and can be accomplished through the collection and analysis of peripheral blood samples, and the quantification and characterization of specific populations of white blood cells[3]. There are many methods described for nonterminal collection of peripheral blood. These include sampling blood from the submandibular plexus, retroorbital sinus, saphenous vein, tail vein, facial vein, jugular vein, etc. [4,5]. Submandibular and retroorbital collection both are invasive and carry the risk of tissue damage. Some other methods routinely involve the collection of greater than 100 µl of blood so that samples can be examined with automated analyzers[4]. Importantly, methods involving anaesthetization or even mouse restraint can be very stressful, which may alter the blood contents [6–8]. In addition to impacting the welfare of the mouse, stress can affect quantities of white blood cells and impact the results of blood assays [1,5]. Tail venipuncture is an alternate low-volume and non-invasive blood collection method that can mitigate stress response in mice by eliminating the need for anesthesia or restraint and reducing time required for the procedure. The low blood volume collected is ideal for longer-term experiments which require regular and continuous monitoring.

This technical report describes a quick, low-stress protocol for collecting blood from the tail vein of aging mice, and for performing total and differential white blood cell (WBC) counts. Türk’s solution is used to obtain a volumetric density of WBCs; Wright-Giemsa stained blood films are used to obtain differential counts and the cell morphology of WBCs. Together, a quantification of differential WBCs can be obtained. Türk’s solution is comprised of acetic acid, which is responsible for the hemolysis of erythrocytes, and a dye to stain the remaining WBCs. A hemocytometer is used to calculate the total WBCs per unit volume. Wright-Giemsa stain is applied onto fixed blood smears for WBC differentiation. WBCs can be separated manually under the microscope through differences in their nuclei shape, cytoplasmic color, and cell shape upon staining. While time-consuming when compared to automated analyzers, manual reading allows both stains to be performed with an extremely small amount of blood. Manual reading of blood films also minimizes errors in characterizing abnormal or less abundant cell types and may adjust for factors such as platelet clumping[7]. The cumulative WBC counts and their differential counts can be monitored over time for any changes, and these changes or certain characterizations can be indicative of disease[7].

## Protocol

### Equipment and materials

General: C57Bl/6 Mice, Microscope, 10, 100 µl pipette tips, Pipette.

Blood Collection: Capillary tubes, heparinized, 22 g needles, 1:18:1 dilution of Clidox-S or another sanitizing agent, isopropyl alcohol or iodine.

Leukocyte Counts: 0.5-ml micro-centrifuge tubes, Türk’s solution, Hemocytometer, Microscope slides, 100% Methanol, Wright-Giemsa stain, Deionized water/phosphate buffer.

### Blood collection

**Figure 1.**
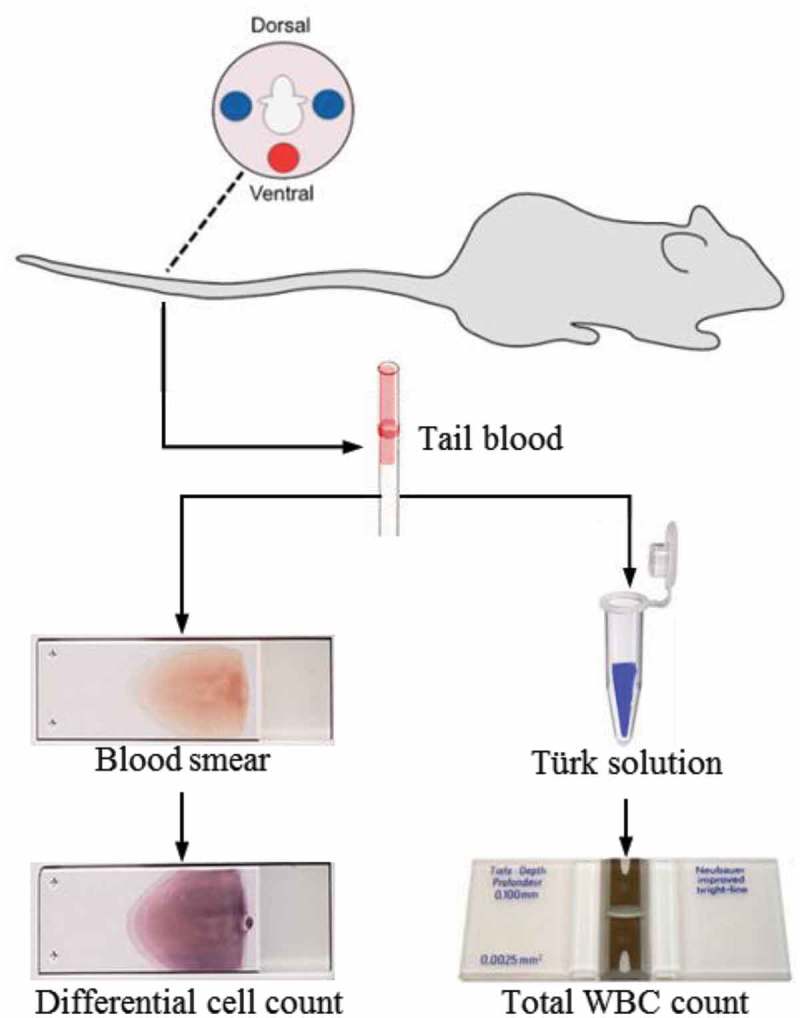
Blood is collected from the tail vein of the mouse, at a blood collection site located down three-fourths of the tail from the base. Approximately 4 µl blood is collected into a heparinized capillary tube. Two-microliter blood is mixed with Turk’s solution for total WBC count. The rest 2 µl blood is used to create blood smear for WBC differentiation. Blue circles: target tail veins, located on both the left and right side of the tail; Red circle: tail artery.

1. Transfer 40 µl of Türk’s solution into a microcentrifuge tube. (Solution volume can be changed based on expected dilution ratio).
2. Use Clidox-S or other sanitizing reagent to cleanse a horizontal surface.
3. Place mouse’s tail on a flat, sanitized horizontal surface, holding the tail steady with one hand.
4. Disinfect the desired venipuncture site on the tail (about two-thirds down the tail, from the tail root) with an isopropyl alcohol towelette or iodine solution.
5. Vertically puncture the tail with a needle. The target tail vein is at approximately three-fourths of the tail from the tail root, located on the left or right sides of the tail (Figure 1).
6. One drop of dark red blood shall bead up after puncture. Collect 4 µl of blood with a capillary tube, which is approximately to the red mark, 0.5 cm from the end of the tube.
7. Once enough blood is collected, wipe off extra blood from the tail with Clidox-wetted paper towel and compress until bleeding stops.
8. Blood should be used for blood smear creation or mixed with Türk’s solution immediately after collection to prevent clotting.

### Blood smear and cell count preparation

1. Transfer 2 µl of blood from the capillary tube using a 10 µl tip into the micro-centrifuge tube containing Türk’s solution. After blood is mixed with Türk’s solution, the mixture should be let sit for at least 30 min before analysis to ensure adequate hemolysis of erythrocytes.
2. Transfer the remaining blood onto a clean slide. With the clean end of another microscope slide, create a blood smear. This blood smear should be fixed in methanol within an hour to avoid cell degradation.

### Total WBC count

1. Pipette 20 µl of the blood-Türk’s solution mixture from the microtube into one of the hemocytometer’s counting chambers. Mix the solution adequately before pipetting as the WBCs tend to settle to the bottom. Wait 1 min for WBCs to settle down.
2. View under 10X microscope, tallying all WBCs in the four corners of the gridded area of the counting chamber[9]. The WBCs shall be a dark purple color.
3. Calculate and record the total while blood cell number per microliter with the following equation:

$$\frac{count\;\#}{4}\times 20\left(dilution\;factor\right)\times 10$$

The dilution factor can be calculated by dividing Türk’s solution volume by blood volume used. Here, the dilution factor is 20, because 2 µl of blood was originally mixed with 40 µl of Türk’s solution.

### Differential WBC count

1. Fix blood smears with methanol for 5 min by immersing the entire microscope slide with the blood smear into methanol.
2. Remove blood smears from methanol and allow to air dry. At this point, blood is fixed onto the slides and staining may be performed at a later time.
3. Use the Wright-Giemsa stain to stain the blood smears:
  - Immerse blood smears in Wright-Giemsa stain for 30 s.
  - Remove from Wright-Giemsa and place blood smears into deionized water for 1–10 min.
  - Wipe back of the slide with a paper towel.
  - Allow to air dry.

**Figure 2.**
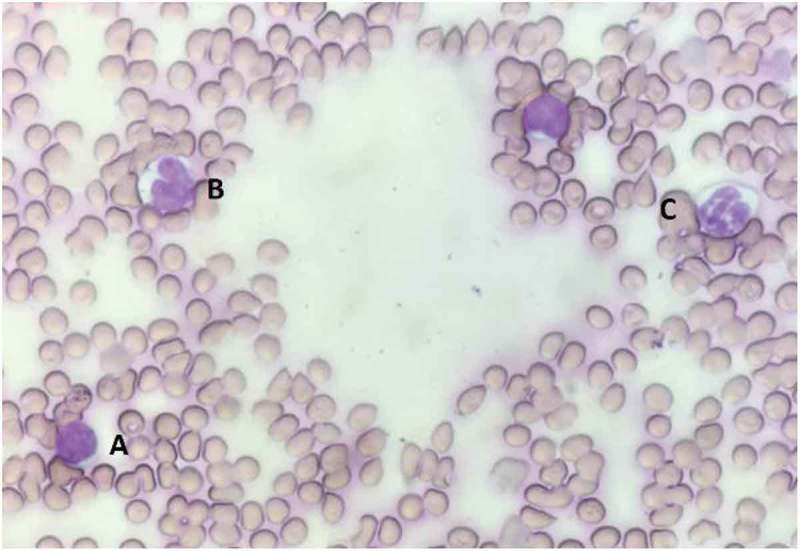
White blood cell differentiation is performed on blood smear with Wright-Geimsa staining. (a) Lymphocyte is characterized by dark purple circular nucleus; (b) Monocyte is characterized by a purple kidney-shaped nucleus with lysosomes in cytoplasm; (c) Neutrophil is characterized by purple multi-lobular nucleus.

Under the microscope at a power sufficient to distinguish the shapes of the stained nuclear bodies (40X~100X), count the first 100 (or 200 or 500 for higher accuracy) WBCs, and categorize them as lymphocytes, neutrophils, monocytes, or other WBCs (Figure 2).

(4) Using total WBC count obtained previously, calculate the differential density of WBCs per microliter for each type of lymphocyte using the following equation.

$$\begin{array}{rl} & Differential\;White\;Blood\;Cell/\mu =\frac{\#\;of\;target\;cell}{\#\;WBC\;counted}\\ & \;\;\;\;\;\;\;\;\;\;\;\;\;\;\;\;\;\;\;\times Total\;White\;Blood\;Cell\;\#/\mu l \end{array}$$

### Serial WBC counts in old C57BL/6 mice

**Figure 3.**
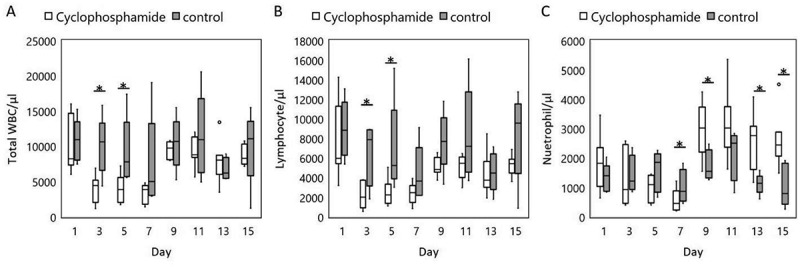
Serial blood collection and WBC counts in 28-month-old C57BL/6 mice given one dose of cyclophosphamide (100 mg/kg IP) provided significant observations. (a) Total WBC; (b) Lymphocytes; and (c) Neutrophils. The control groups showed no significant variation, while there was a significant decrease in the cyclophosphamide groups. N = 6–8 mice per cohort,*p < 0.05 by student t-test.

C57Bl/6 male mice, 28 months of age, were injected with 100 mg/kg cyclophosphamide, and blood collected once every 2 days for 2 weeks. For neutrophil counts, the control group (n = 6) showed relative stable neutrophil level with no statistical difference, while the cyclophosphamide group (n = 8) had a significant decrease followed by a rebound (Figure 3). The results indicate our method is able to safely collect blood to detect drug-induced WBC changes without causing detectable adverse effects or stress to the old mice.

## Conclusions

This method can be performed once every 2 days for extended periods without causing any observable changes in the mouse. Handling the mouse before the first blood collection will decrease anxiety to help avoid any introductory stress. With the appropriate puncture technique and correct needle size, more than 4 µl of blood may be collected directly after puncture. If the needle misses the tail vein, additional blood can be squeezed out by gently applying pressure around the pierced spot. Re-puncturing the tail is not recommended and should be avoided if possible, as this may cause stress. The mouse should stop bleeding within 1 min unless the artery is broken. In such cases, hemostasis should be applied. Since the WBC amount and composition can be impacted by stress, it is important to avoid other unexpected stressors during the experiment. One particularly stressful event includes cage changing, which may affect the WBC readings if performed within the same day[10]. It is also important, if collecting serial blood samples, to take blood at the same time every day, as blood contents may change throughout the day. The blood obtained from the tail should not be compared to other methods as the blood collection site can affect the results[11].

This procedure can easily be modified to suit the needs of specific studies, for example:

1. The dilution factor can be changed based on the target model. For example, more Türk’s solution can be added for leukemia models to reduce the counting difficulty.
2. Extra blood can be collected for other types of analysis by gently squeezing the tail. For total and differential cell count only 4 µl is suggested.
3. To increase the amount of bleeding, a larger needle can be used. Moving the puncture point toward the tail root can increase bleeding but also the difficulty of finding the vein.
4. To collect arterial blood, twist the tail axially by 90°.
5. If only the blood smear is needed, a 28G needle may be used, and the collection can be done more frequently.
6. For Wright-Giemsa stain, a longer staining time can deepen the color to study cell morphology. But overstaining will cause the color overlay between cytoplasm and nucleus.
7. Staining with solutions other than Wright-Giemsa can also be performed on the blood smear to gather other information.

Compared to other blood collection techniques, this method only requires small amounts of blood and short handling times. As mice are sensitive to stress, invasive tests can affect or even nullify the experimental results. It is essential to develop low-stress methods to obtain higher accuracy.
